# Development and external validation of a prediction model for digit replantation failure after traumatic amputations based on a prospective multicenter cohort

**DOI:** 10.1097/JS9.0000000000001145

**Published:** 2024-02-13

**Authors:** Tao Gao, Bingbo Bao, Junqing Lin, Maoyuan Tian, Lei Xia, Haifeng Wei, Qianying Cai, Hongyi Zhu, Xianyou Zheng

**Affiliations:** a Department of Orthopaedic Surgery, Shanghai Sixth People’s Hospital Affiliated to Shanghai Jiao Tong University School of Medicine, Shanghai; b Department of Orthopaedic Surgery, 80 PLA Hospital Shandong; c Department of Hand Surgery, Xi’an Honghui Hospital, Nanshaomen, Xi’an, Shaanxi, People’s Republic of China

**Keywords:** amputation, replantation, traumatic, nomograms

## Abstract

**Background:**

Failure of digit replantation after traumatic amputation is difficult to predict. The authors aimed to develop a prognostic model to better identify factors that better predict replantation failure following traumatic digit amputation.

**Materials and methods:**

In this multicenter prospective cohort, the authors identified patients who had received digit replantation between 1 January 2015 and 1 January 2019. Univariable and multivariable analyses were performed successively to identify independently predictive factors for failure of replanted digit. To reduce overfitting, the Bayesian information criterion was used to reduce variables in the original model. Nomograms were created with the reduced model after model selection. This model was then internally validated with bootstrap resampling and further externally validated in validation cohort.

**Results:**

Digit replantation was failed in 101 of 1062 (9.5%) digits and 146 of 1156 digits (12.6%) in the training and validation cohorts, respectively. The authors found that six independent prognostic variables were associated with digit replantation failure: age, mechanism of injury, ischemia duration, smoking status, amputation pattern (complete or incomplete), and surgeon’s experience. The prediction model achieved good discrimination, with concordance indexes of 0.81 (95% CI: 0.76–0.85) and 0.70 (95% CI: 0.65–0.74) in predicting digit failure in the training and validation cohorts, respectively. Calibration curves were well-fitted for both training and validation cohorts.

**Conclusions:**

The proposed prediction model effectively predicted the failure rate of digit replantation for individual digits of all patients. It could assist in selecting the most suitable surgical plan for the patient.

Highlights

- This multicenter prospective cohort is the most large-scale one on finger replantation to date.
- A prediction model was well developed based on age, mechanism of injury, duration of ischemia, smoking status, amputation pattern, and surgical experience of surgeon.
- After validation, this model can effectively predict the failure rate of digit replantation for individual digits of all patients.

## Background

Approximately 1% of all traumatic injuries treated in the emergency room are limb amputations, and of these, 90% involve traumatic digit amputations^1–4^, mostly in young, active working population. With the global acceleration of industrialization, digit amputation exerts huge economic burden to society and patients, and the average direct and indirect expense of every injury reached $30 754^2,5,6^. Moreover, traumatic digit amputations severely affect both physical functioning and psychological health, such as depression, anxiety, and PTSD^7,8^.

To date, treatment of traumatic digit amputation mainly includes procedures such as digit replantation and revision amputation. While revision amputation is less costly and less complex, replantation is still an important treatment for patients with traumatic digit amputation, in that it can partially restore the function and appearance of the affected finger^3^. However, this sophisticated surgery requires highly experienced hand surgeons and patients require long or extended hospitalization after surgery^9,10^. Owing to microsurgical advancements, the success rate of digit replantation reached more than 80%^11–13^. But there is still a certain probability of failure, especially in patients with some adverse influencing factors^14^. Once replantation fails, patients will suffer unacceptable consequences, such as a secondary revision amputation, higher subsequent treatment costs, prolongment of the treatment, delay in returning to work, and even a shorter finger than received initial revision amputation in ED surgery^15,16^.

According to current studies, many factors affect the failure rate of digit replantation, including the patient’s demographic and clinical characteristics (e.g. age, smoking status, presence or absence of underlying diseases such as hypertension and diabetes, etc.); injury mechanism; duration of warm ischemia; hospital census at admittance; and surgeon proficiency^17–19^. However, for the limitation of sample size, no study revealed the ratio of every single factor contributing to the failure rate of replantation to date. Therefore, it would be useful to develop a simple, easy-to-use model that clinicians could apply to predict the failure rate of replantation for each affected finger by integrating different influence factors and to decide whether replantation or revision amputation should ultimately be performed. Having this information in mind, patients and surgeons can weigh the risks of different treatments (e.g. replantation versus no replantation), their respective costs and time requirements, as well as the risk of secondary surgery.

As a developing country with a highly developed industry, China has a huge group of manual workers, which suffered from machine-related traumatic digit amputation. Due to the traditional concept and universal coverage of work-related insurance, patients have a higher replantation rate in China (30–44%) than that in US (18%)^9,18,20^. Therefore, this study, conducted in three centers, which perform a sufficiently high-volume of digit replantation and distributed in different parts of China, provided a large sample size to develop and validate a model to predict the failure of digit replantation. The three centers contributing data to the development and validation of the nomogram are ideal sources, because they distributed in different part of the country of study, making the proposed nomogram more generalizable.

## Materials and methods

### Study design and patient population

This is a multicenter prospective cohort study which enrolled all patients with digital traumatic amputation^20^. We first identified patients who received digit replantation at anyone of the three emergency trauma centers between 1 January 2015 and 1 January 2019, from three hospitals in China. Exclusion criteria were having single or multiple organ failure (*n*=7), peripheral artery disease (*n*=15), additional arterial trauma in the ipsilateral arm or forearm (*n*=145), younger than 18 years old (*n*=51), contraindications for digit replantation (*n*=25), refuse to participate or unable to understand Mandarin Chinese (*n*=988). Patients’ demographic and basic clinical data were collected and recorded in a central electronic data capture system, which was accessible only by study personnel from all three centers. Patient data were de-identified and protected by privacy safeguards appropriate to local regulations.

Patients’ demographic data, including age, sex, smoking status (yes/no), and comorbidities (e.g. hypertension and diabetes mellitus) were self-reported. Patients also self-reported the approximate time and primary cause or mechanism of digit amputation, which we classified as either crush, saw, avulsion, or cut trauma. The surgeon evaluated and recorded the time of arterial revascularization and whether it was a complete/incomplete amputation; we called this latter categorization the pattern of amputation (e.g. Table 1). Radiographs and consultation notes were used to determine the digit(s) involved and the Tamai level(s) of the injury.

**Table 1:** Demographic and clinical characteristics of training cohort grouped by outcome^a^.

	Replantation outcome		
	Success (*n*=961)	Failure (*n*=101)	*P* ^b^
Mean age (±SD)	36.2±12.0	42.3±11.7	<0.001
Sex			
Male	831 (86.5)	86 (85.1)	0.712
Female	130 (13.5)	15 (14.9)	
Tamai^c^			
V	130 (13.5)	13 (12.9)	0.03
IV	271 (28.2)	18 (17.8)	
III	293 (30.5)	46 (45.5)	
II	147 (15.3)	13 (12.9)	
I	120 (12.5)	11 (10.9)	
Mechanism of injury			
Saw	421 (43.8)	44 (43.6)	<0.001
Crush	137 (14.3)	17 (16.8)	
Avulsion	118 (12.3)	29 (28.7)	
Blade	285 (29.7)	11 (10.9)	
Ischemia duration (mean hours±SD)	8.6±2.9	10.8±3.2	<0.001
Smoking status			
Smoker	335 (34.9)	56 (55.4)	<0.001
Nonsmoker	626 (65.1)	45 (44.6)	
Amputation pattern			
Complete	701 (72.9)	91 (90.1)	<0.001
Incomplete	260 (27.1)	10 (9.9)	
Surgeon’s experience^d^			
Trainee	186 (19.4)	35 (34.7)	<0.001
Specialist	775 (80.6)	66 (65.3)	
Education level			
Less than college graduate	817 (85.0)	83 (82.2)	0.451
College graduate or higher	144 (15.0)	18 (17.8)	
Comorbidities			
Hypertension	139 (14.5)	17 (16.8)	0.523
No hypertension	822 (85.5)	84 (83.2)	
Diabetes mellitus	87 (9.1)	10 (9.9)	0.778
No diabetes mellitus	874 (90.9)	91 (90.1)	

a Data are presented as *n* (%), unless otherwise noted.

b Mann–Whitney tests or Fisher’s exact tests were used, depending on whether the variables were continuous or categorical.

c Tamai level of injury as described by Yoshimura (2003).

d Specialists were defined operationally as surgeons who performed 50 or more-digit replantations in his or her career, and trainees were defined as surgeons who performed 49 or fewer digit replantations.

The experience of the surgeon who performed the digit replantation for each patient was classified as a either trainee level or specialist level, according to the number of digital replantation performed in his or her career. Specialists were defined operationally as surgeons who performed 50 or more-digit replantation in his or her career, and trainees were defined as surgeons who performed 49 or fewer digit replantation.

This study was approved by the ethics committees of Shanghai Sixth People’s Hospital Affiliated to Shanghai Jiao Tong University School of Medicine, 80 PLA Hospital, and Xi’an Honghui Hospital. The study has been reported in line with the strengthening the reporting of cohort studies in surgery (STROCSS) criteria^21^ and conformed with the reporting TRIPOD guidelines for developing, validating, or updating a prediction model^22^. Full written informed consent was obtained from all participants.

### Surgery and postoperative procedures

All replantation operations were performed in emergency operating rooms soon after the injury. Postoperatively, the patient was kept in a still recumbent position, the affected limb was kept warm by applying incident light from a heat lamp, and the affected limb was elevated above the heart or slightly higher to increase circulation^23^. The blood supply to the affected finger was closely monitored, and the patient was managed according to standard vascular crisis procedures. Re-exploration after initial surgery, if needed, was performed by specialists with high expertise in microsurgery. All patients were followed up for at least 1 month. Failure was defined as necrosis of the replanted finger, which required revision amputation or a flap cover of the skeleton^17^.

### Statistical analysis

All analyses were performed using SPSS (IBM Corp. Released 2016. IBM SPSS Statistics for Windows, Version 24.0. Armonk, NY: IBM Corp.) and R software version 3.5.3 (R Core Team. Released 2019. R: A Language and Environment for Statistical Computing. R Foundation for Statistical Computing. https://www.R-project.org). Variables with continuous and categorical values were evaluated using the Mann–Whitney test or Fisher’s exact test, respectively. The significance of each variable in the training cohort was first assessed using univariate analysis. When a variable yielded a *P*-value of <0.2 in the univariate analysis, it was included in the subsequent binary analysis. The Bayesian information criterion was then used to reduce the number of variables introduced into the model. The nomogram was then developed based on the size of the resulting β coefficients of the multivariate logistic regression analysis and by using the rms package of R software (version 3.4.1). The variable with the largest β coefficient was assigned 100 points, and remaining variables were proportionally transformed along a 0-to-100-point scale according to the size of their β coefficients. For internal validation, the discriminative performance of the nomogram was measured using the concordance index (C-index). Internal calibration was performed using 1000 bootstrap samples of the training cohort data to assess any overfitting bias. For external validation, discriminative performance, and calibration were assessed likewise using the validation cohort data. In all statistical analyses, a *P*-value of <0.05 was considered to be statistically significant.

## Results

**Figure 1:**
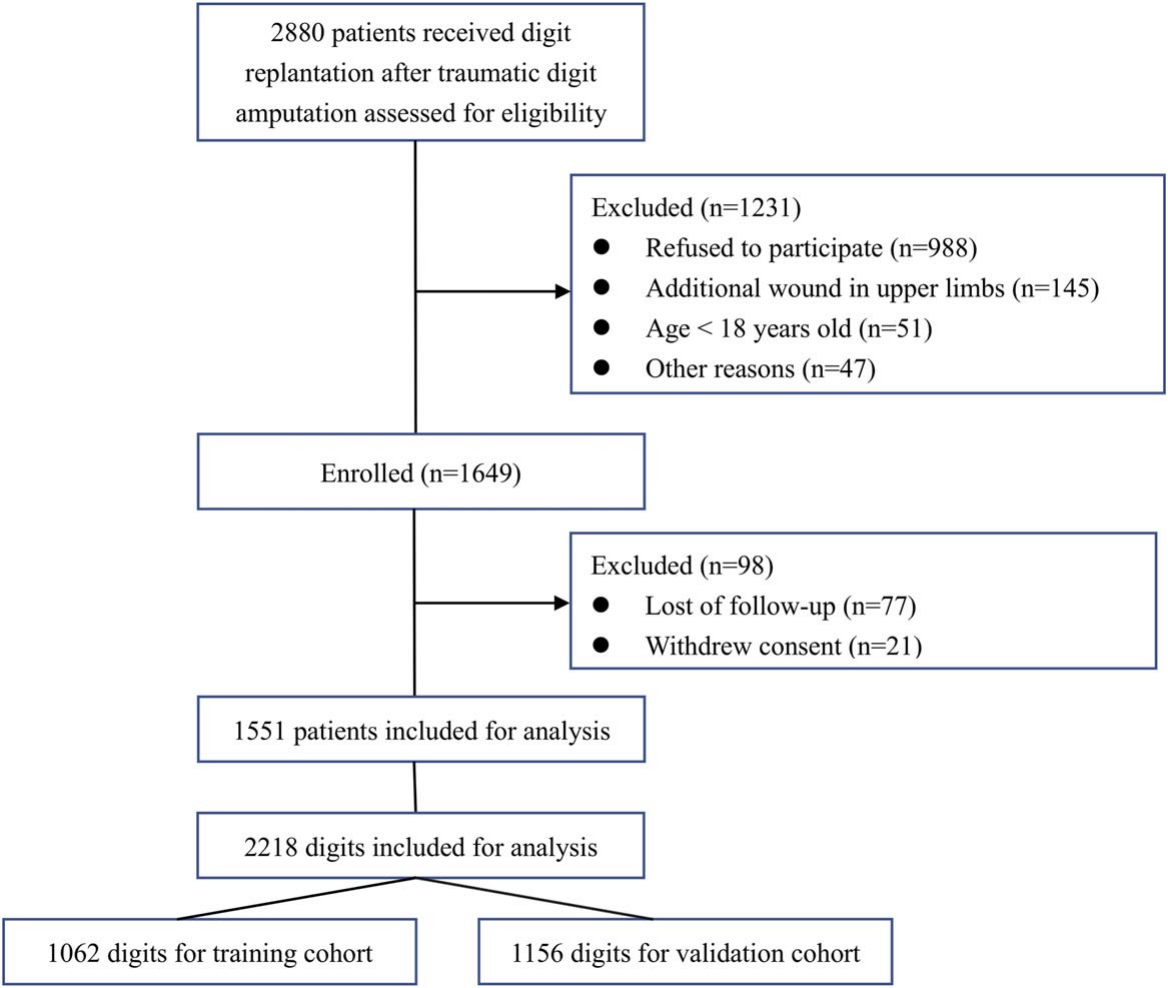
Study screening and enrollment.

For training and validation cohorts, respectively, we included 745 and 806 patients with 1062 and 1156 replanted digits, respectively, as shown in Figure 1. Overall failure of replantation was 101 of 1062 (9.5%) digits in the training cohort and 146 of 1156 digits (12.6%) in the validation cohort.

### Univariable analysis of prognostic factors

Table 1 presents and compares demographic and clinical characteristics of the training cohort based on the replanted digits. All variables were evaluated using univariable analyses according to success or failure of replantation. In the training cohort, the success and failure groups differed significantly along several variables: age, level of amputation (Tamai level), mechanism of injury, duration of ischemia, smoking status, amputation pattern, and surgeon’s experience. There were no statistically significant differences between the success and failure groups in demographic characteristics, comorbidities, or educational level.

### Model selection Bayesian information criterion

The variables of patient age, level of amputation (Tamai level), mechanism of injury, duration of ischemia, smoking status, amputation pattern, and surgeon’s experience yielded *P*-values of <0.2 and thus were included in the binary analysis. To avoid overfitting the model, we used the Bayesian information criterion to reduce the number of variables. Age, mechanism of injury, duration of ischemia, smoking status, amputation pattern, and surgeon’s experience were retained in the final reduced model; these variables are presented in Table 2.

**Table 2:** Results of binary logistic regression analysis to construct nomogram.

	Odds ratio	95% CI	*P*
Age	0.966	0.960–0.972	＜0.001
Mechanism of injury			
Avulsion	Reference		
Crush	1.412	1.124–1.774	0.003
Saw	2.139	1.778–2.572	＜0.001
Blade	6.697	5.024–8.928	＜0.001
Ischemia duration	0.799	0.777–0.821	＜0.001
Smoking status			
Smoker	Reference		
Nonsmoker	1.631	1.399–1.900	＜0.001
Amputation pattern			
Complete	Reference		
Incomplete	3.336	2.656–4.190	＜0.001
Surgeon’s experience			
Trainee	Reference		
Specialist	2.406	2.039–2.840	＜0.001

### Development and validation of prognostic model for replantation failure

**Figure 2:**
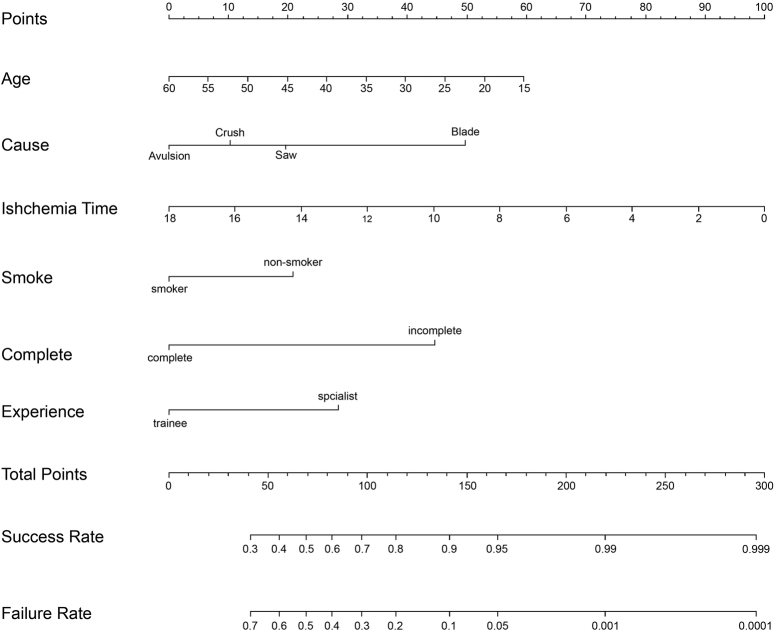
Proposed model for estimating failure rate of digit replantation after traumatic digit amputation. To use the prediction, first, the surgeon determines the subscore of each variable by finding the patient’s value on the corresponding scale and then drawing a vertical line up to the Points scale to obtain the number of points for that variable. This is done for each variable. Second, the surgeon calculates the sum of the variable subscores. Finally, the surgeon finds the sum of the variable subscores on the Total Points scale, and then draws a vertical line (index line or isopleth) from the total points scale down to the lower line (failure rate) of the model to determine the predicted probability of failure.

**Figure 3:**
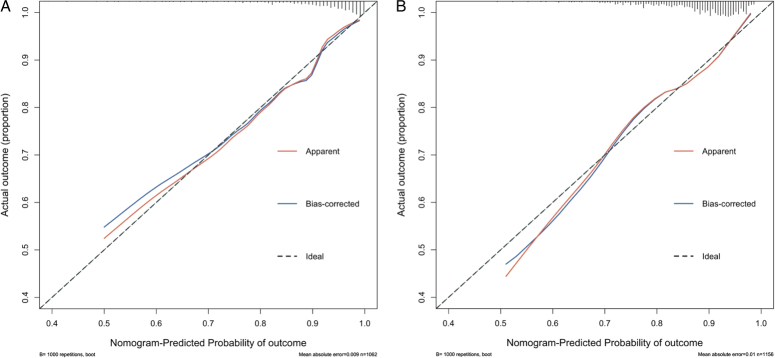
Bootstrap analysis results to determine prediction accuracy of model for failure rate of digit replantation. Calibration curve of the model for (A) training and (B) validation cohorts in estimating failure rate. The prediction model was validated with the internal data from the training cohort, and then externally evaluated from data of the validation cohort. Apparent (or actual) accuracy, bias-corrected accuracy, and ideal accuracy are plotted. Analysis used 1000 bootstrap samples.

The proposed model for failure rate prediction after digit replantation was constructed using the independently associated variables evaluated and presented in Table 2. These variables and their associated scales are shown in Figure 2. We used the bootstrap validation method to internally and externally validate the prediction accuracy of this prediction model on the training and validation cohorts, respectively (Fig. 3).

The prediction model had good accuracy^24^ in predicting the failure of digit replantation. The C-index for the model was 0.81 (95% CI: 0.76–0.85) for the training cohort and 0.70 (95% CI: 0.65–0.74) for the validation cohort. These were consistent with calibration plots, which showed good agreement for predicted and actual failure rate (Fig. 3). For external validation, although the validation cohort was recruited from the two hospitals geographically distant from the hospital from which the training cohort was recruited, there were no significant differences in many baseline and treatment characteristics between the two groups (Table 3).

**Table 3:** Comparison of demographic and clinical characteristics of training and validation cohorts^a^.

	Training (*n*=1062)	Validation (*n*=1156)	*P*
Mean age (±SD)	36.8±12.1	37.5±12.6	0.151
Sex			
Male	917 (86.3)	983 (92.6)	0.396
Female	145 (13.7)	173 (16.3)	
Tamai level			
V	143 (13.5)	164 (15.4)	0.853
IV	289 (27.2)	333 (31.4)	
III	339 (31.9)	348 (32.8)	
II	160 (15.1)	171 (16.1)	
I	131 (12.3)	140 (13.2)	
Mechanism of injury			
Saw	465 (43.8)	502 (47.3)	0.138
Crush	154 (14.5)	208 (19.6)	
Avulsion	147 (13.8)	148 (13.9)	
Blade	296 (27.9)	298 (28.1)	
Ischemia duration (mean hours ±SD)	7.2±3.5	7.0±3.7	0.172
Smoking status			
Smoker	391 (36.8)	430 (40.5)	0.86
Nonsmoker	671 (63.2)	726 (68.4)	
Amputation pattern			
Complete	792 (74.6)	845 (79.6)	0.44
Incomplete	270 (25.4)	311 (29.3)	
Surgeon’s experience			
Trainee	221 (20.8)	255 (24)	0.501
Specialist	841 (79.2)	901 (84.8)	
Patient education level			
Less than college graduate	900 (84.7)	984 (85.1)	0.805
College graduate or higher	162 (15.3)	172 (14.9)	
Comorbidities			
Hypertension	156 (14.7)	173 (15.0)	0.855
No hypertension	906 (85.3)	983 (85.0)	
Diabetes mellitus	97 (9.1)	113 (9.8)	0.606
No diabetes mellitus	965 (90.9)	1043 (90.2)	

a Data are presented as *n* (%), unless otherwise noted. Mann–Whitney tests or Fisher’s exact tests were used, respectively, for continuous and categorical variables.

## Discussion

There are many factors that affect the failure of digit replantation, including demographic characteristics (e.g. age, smoking status, and comorbidities); injury characteristics (e.g. mechanism, pattern of amputation, duration of ischemia, etc.); and replantation surgery characteristics (e.g. surgeon’s experience and quality of vascular anastomosis)^25^. A huge challenge for hand surgeons in selecting a treatment plan for an individual amputated digit is to estimate accurately the likelihood of replantation failure for each patient. To date, no effective tools exist to assist surgeons in evaluating the failure of digit replantation. Nomograms are widely used in surgical planning and many other medical fields^21–23^. With a nomogram, physicians can visually estimate the individual probability of a specific clinical event by integrating diverse prognostic variables. With the nomogram and consideration of patients’ financial resources, clinical decisions can be made more rationally together with patients and their treating surgeons.

In the current prospective multicenter study, we developed and validated an evidence-based nomogram that accurately predicts the failure rate of digit replantation in patients with digit amputation(s). Internal and external validation demonstrated that the nomogram was a strong model and had good accuracy in predicting outcome. We found that patient age, mechanism of injury, duration of ischemia, smoking status, amputation pattern (complete or partial), and the surgeon’s experience were good predictors of replantation failure.

When patients arrive in the emergency room with a traumatic digit amputation, typically, hand surgeons estimate the failure rate of replantation. Simultaneously, the surgeon evaluates available information about possible complications, treatment costs, and expectation for recovery of function. Then, the surgeon and patient can weigh all these considerations and determine together what kind of treatment plan to adopt. Some predictors, such as age, mechanism of injury, smoking status, and amputation pattern are unchangeable after traumatic digit amputation, while others, like duration of ischemia and surgeon’s experience, have the potential of being favorably modified and thus can potentially affect the failure rate of replantation.

Whether the duration of ischemia affects replantation survival remains controversial^17,26,27^. In our study, the duration of ischemia occupies a considerable proportion of points in the nomogram, as it can be an excellent predictor of the failure rate of a replanted digit. Consistent with the findings of previous studies^12,18,19,28^, our nomogram indicated that a surgeon’s experience is positively correlated with the failure rate of finger digit replantation, and it has a higher weight ratio than smoking status. Therefore, this should be fully considered in future clinical decision-making to patients with traumatic digit amputation. However, its impact on decreasing points of the failure rate is greatly reduced after it reaches a certain level (such as 140, which corresponds to about a 10% failure rate), according to the nonlinear correlation between total points and failure rate indicated by Figure 2. Thus, for a young, nonsmoking patient with an incompletely amputated digit caused by a knife blade injury and who was rushed to the emergency room soon after the injury, the failure rate of digit replantation would be still low even if performed by a trainee. For an older, smoking patient with a completely amputated digit caused by an avulsion injury and having a long-duration ischemia, the failure rate would decrease significantly if performed by a surgeon at a specialist level of experience compared with a trainee.

For a hospital that has a high-volume of digit replantation, several patients with traumatic digit amputations may arrive at the emergency room simultaneously. The overall failure rate of digit replantation would decrease if hand surgeons and patients were matched according to the surgeons’ experience and the patients’ complexity of injury, with experienced surgeons being matched with patients having the more complex injuries and trainees being matched to those with less complex injuries, etc. In cases where patients arrive at a hospital that has a low-volume of digit replantation, patients must weigh whether to leave that hospital and go to a high-volume hospital where they can be treated by a specialist (thus increasing the duration of ischemia) or to remain in the low-volume hospital and undergo digit replantation immediately (thus decreasing the duration of ischemia). Our prognostic model was shown to successfully evaluate the failure rate by integrating all these factors. Also, it can guide patients and surgeons in decision-making about the optimal treatment plan for their individual situation.

### Limitations

This study has some limitations. Firstly, multiple future prospective studies are needed to validate and possibly calibrate our model to improve its predictive accuracy across different situations. This is especially the case when intraoperative factors (e.g. the number and quality of vascular anastomosis^29^) and postoperative factors (e.g. the administration of antithrombotic therapies^30^) were not considered in the present study. Secondly, our study was conducted in three hospitals that have a high annual volume of replantation and the surgeons there may have more experience with replantation than at low-volume hospitals, biasing the test-bed for our model. Thus, the prediction model needs to be validated in low-volume hospitals. Thirdly, the clinical usefulness of this model is still unknown despite its high predictive accuracy. It can be assessed by designing a future randomized controlled study, in which patients are randomly assigned to nomogram-assisted or non-nomogram-assisted groups and patient outcomes are compared. Finally, our prediction model cannot be used alone to guide clinical decisions because it did not consider the functional prognosis.

## Conclusion

The novel model developed for patients with traumatic digit amputation(s) can effectively predict the failure of digit replantation for individual digits of all patients. While considering the patient’s economic status, nature of work, etc. physicians and patients can use the prediction model to jointly decide on the most suitable surgical plan for the patient. Finally, the results of this study may also be used to guide the development and validation of nomograms for other types of limb amputations.

## Ethical approval

The study was approved by the Ethics Committee of Shanghai Sixth People’s Hospital Affiliated to Shanghai Jiaotong University School of medicine. Approval No: 2013-KY-029. The study was approved by the Ethics Committee of 80 PLA Hospital and Xi’an Honghui Hospital.

## Sources of funding

This study was supported by National Natural Science Foundation of China (Grant 82002324, Hongyi Zhu; Grant 81974331, Xianyou Zheng); Clinical Technology Innovation Project of Shanghai Hospital Development Center (Grant SHDC12019X06, Xianyou Zheng); the Clinical Research Project of Shanghai Municipal Health Commission (Grant 20194Y0254, Hongyi Zhu).

## Author contribution

X.Z.: had full access to all the data in the study and take responsibility for the integrity of the data and the accuracy of the data analysis; T.G., B.B., and J.L.: share co-first authorship, and X.Z., H.Z., and Q.C.: share co-senior authorship; X.Z. and H.Z.: concept and design; T.G., B.B., J.L., M.T., L.X., and H.W.: acquisition, analysis, or interpretation of data; X.Z. and H.Z.: critical revision of the manuscript for important intellectual content; H.Z. and Q.C.: statistical analysis; X.Z. and H.Z.: obtained funding; X.Z., Hongyi Zhu and Qianying Cai

## Conflicts of interest disclosure

All authors declares no competing interests.

## Research registration unique identifying number (UIN)

Trial registry: Chinese Clinical Trial Registry.

Registration number: ChiCTR1900026268.

## Guarantor

Xianyou Zheng.

## Data availability statement

No additional data are available.
